# A patient with recurrent palpitations and unusual anatomy

**DOI:** 10.1007/s12471-020-01403-3

**Published:** 2020-03-19

**Authors:** C. J. M. Lawson, A. D. Margulescu, J. Barry

**Affiliations:** Department of Cardiology, Morriston Regional Cardiac Centre, SA6 6NL Swansea, UK

## Answer

**Fig. 1:**
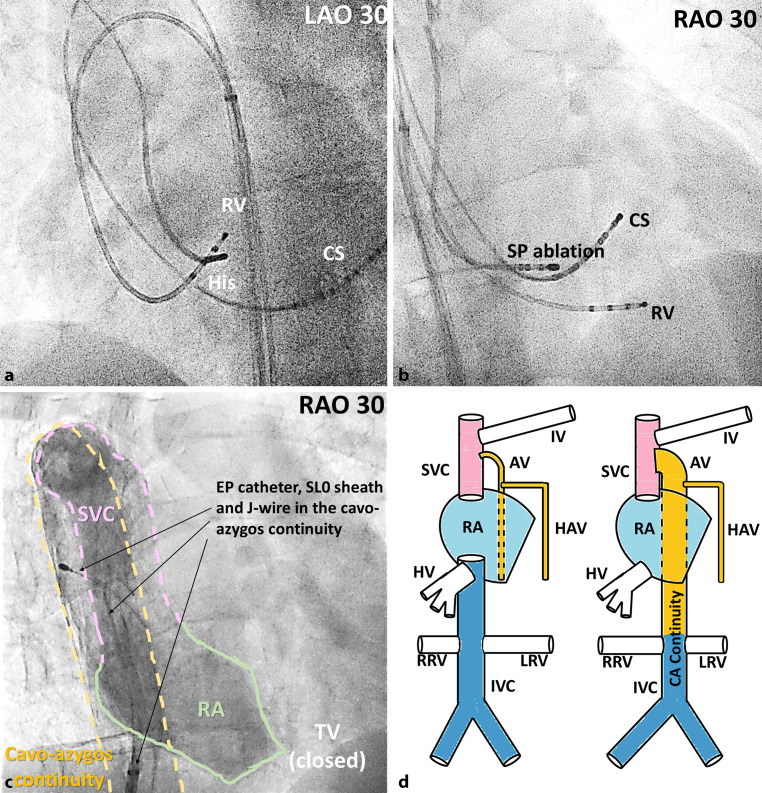
Inferior vena cava atresia with cavo-azygos (CA) continuity accounts for the unusual placement of the electrophysiology catheters from the right inguinal region into the heart. **a** LAO 30, left anterior oblique 30 degrees view; **b** RAO 30, right anterior oblique 30 degrees view; **c** Contrast venogram helped deliniate the anatomical structures through which the electrophysiology catheter and sheaths were advanced from the right inguinal region; **d** Schematic representation of normal venous anatomy (*left*) vs. CA continuity (*right*). *CS* coronary sinus, *RA* right atrium *RV* right ventricle *SP* slow pathway *TV* tricuspid valve, *SVC* superior vena cava, *IV* innominate vein, *AV* azygos vein, *HAV* hemiazygos vein, *HV* hepatic veins, *RRV* right renal vein, *LRV* left renal vein

Complete atresia of the inferior vena cava (IVC) with cavo-azygos (CA) continuity was diagnosed by contrast venography performed using a long sheath (SL0, Abbott Medical, USA) (Fig. 1a). Following the CA vein route, a decapolar and a quadripolar catheter were advanced into the superior vena cava, right atrium, and then into the coronary sinus and right ventricle, respectively (Fig. 1b). However, the right subclavian vein needed to be used to map the triangle of Koch with the ablation catheter, due to better reach and stability at this region compared with the CA route (Fig. 1c).

IVC atresia with CA continuity is a rare congenital anomaly that results from lack of interruption of the right cardinal vein at the level of the diaphragm during embryological development [1]. As a result, the intrahepatic trajectory of the IVC is not formed, and the hepatic veins will drain separately into the right atrium. Fig. 1d shows a schematic representation of normal venous anatomy vs. CA continuity. CA continuity may be associated with more extensive embryological abnormalities, such as the heterotaxy syndrome (abnormal arrangement of internal organs across the left-right axis of the body) [2]. In our patient, chest X‑ray, abdominal ultrasound and echocardiogram revealed normal internal organ arrangement.

For electrophysiology procedures, IVC atresia with CA continuity can cause significant challenges, especially if left atrial access is required, because the impossibility of performing transseptal puncture through the usual inferior approach [3, 4]. However, right-sided ablations (including ablation of atrioventricular nodal re-entrant tachycardia) can be performed with small variations of standard techniques, by looping the catheters back into the right cardiac chambers through the superior vena cava, as demonstrated in this case.
